# A Serum Metabolite Classifier for the Early Detection of Type 2 Diabetes Mellitus-Positive Hepatocellular Cancer

**DOI:** 10.3390/metabo12070610

**Published:** 2022-07-01

**Authors:** Lin-Lin Cao, Yi Han, Lin Pei, Zhi-Hong Yue, Bo-Yu Liu, Jing-Wen Cui, Mei Jia, Hui Wang

**Affiliations:** 1 Department of Clinical Laboratory, Peking University People’s Hospital, Beijing 100044, China; caoll@bjmu.edu.cn (L.-L.C.); 1610113144@bjmu.edu.cn (Y.H.); peilin@bjmu.edu.cn (L.P.); yue_zhihong@pkuph.edu.cn (Z.-H.Y.); jiameipku@163.com (M.J.); 2 Department of Pharmacy, Peking University People’s Hospital, Beijing 100044, China; liuboyu0522@126.com; 3 SCIEX Analytical Instrument Trading Co., Shanghai 200335, China; jingwen.cui@sciex.com

**Keywords:** hepatocellular cancer, type 2 diabetes mellitus, metabolomics, metabolite classifier, benzoic acid, creatine, citrulline

## Abstract

Type 2 diabetes mellitus (T2DM) has been identified as an independent risk factor for hepatocellular cancer (HCC). However, there are no ideal biomarkers for the surveillance and early detection of HCC in the T2DM population at present. In this study, we aimed to explore novel metabolite biomarkers for T2DM-positive [T2DM(+)] HCC by metabolomic analysis. At first, many serum metabolites were found dysregulated in T2DM(+) HCC patients in untargeted metabolomic analyses. Targeted metabolite analyses confirmed that serum benzoic acid and citrulline were increased, and creatine was decreased in T2DM(+) HCC compared to the T2DM group. A metabolite classifier including benzoic acid, creatine, and citrulline was identified as a novel biomarker for the diagnosis of T2DM(+) HCC, with an area under the ROC curve (AUC) of 0.93 for discriminating T2DM(+) HCC patients from T2DM patients. In addition, the metabolite classifier detected small-size (AUC = 0.94), early-stage (AUC = 0.94), and AFP-negative (AUC = 0.96) tumors with high sensitivity and specificity. The combination of this metabolite classifier and AFP might be useful in the surveillance and early detection of HCC in the T2DM population. In conclusion, this study establishes a novel diagnostic tool for T2DM(+) HCC.

## 1. Introduction

Primary liver cancer is the sixth most frequently occurring cancer and the third most common cause of cancer death in the world, which seriously threatens human health. Hepatocellular cancer (HCC) is the dominant type of primary liver cancer, comprising 75–85% of all cases [1,2]. Though significant progress has been achieved in the understanding of the risk factors and molecular mechanisms of HCC, and reasonable approaches to prevention, surveillance, and early detection have also been developed over the past few decades, the majority of HCC cases are still diagnosed at an advanced stage [3]. It has been reported that early-stage tumors are suitable for potentially curative therapies, and the prognosis is much better than that of advanced-stage tumors [4,5]. Therefore, the surveillance of high-risk populations and the early diagnosis of tumors are critical for improving the prognosis of HCC patients. 

It has been widely known that chronic hepatitis B virus (HBV) and hepatitis C virus (HCV) infection remain the most important risk factors for HCC at present. However, their importance appears to be declining due to the HBV vaccination of newborns and the effective treatments for both HBV and HCV infections globally [2]. Accumulating evidence suggests that type 2 diabetes mellitus (T2DM) is an independent risk factor for HCC as well [6,7,8,9]. T2DM is a metabolic disorder characterized by hyperglycemia, impaired insulin secretion, and insulin resistance and represents the most common type of diabetes (accounting for over 90% of all cases) [10]. It has been demonstrated that the presence of T2DM is associated with an increased risk of the incidence and mortality of cancer, especially HCC [11,12]. Due to the current T2DM pandemic [13,14], it is essential to monitor the T2DM population to reduce the risk of HCC. However, there is no sensitive and specific biomarker for HCC surveillance in T2DM patients at present. Serum alpha-fetoprotein (AFP) is a widely used HCC biomarker in clinical practice, but its diagnostic accuracy is not satisfactory [15]. Some other circulating biomarkers, such as genetic mutations, microRNAs, and dysregulated DNA methylation, have been shown to have the potential as candidate biomarkers for the early detection of HCC [3,16], but they are not specific for T2DM-positive [T2DM(+)] HCC and are not suitable for HCC surveillance in the T2DM population. Therefore, it is critical to explore novel biomarkers for the surveillance and early detection of HCC in this group. 

Metabolomics, which focuses on the comprehensive assessment of small molecule metabolites, has emerged as a powerful tool for the discovery of novel biomarkers [17]. The liver is an important metabolic center that performs a diverse array of biochemical reactions to maintain the body’s metabolic homeostasis. Undoubtedly, the development of HCC impairs the biochemical functions of the liver, accompanied by changes in the levels of many small molecule metabolites [18]. Therefore, metabolomic analysis is capable of determining dysregulated metabolites to explore novel biomarkers for HCC surveillance and diagnosis [19]. Several studies on HCC metabolomics have been conducted using various kinds of specimens, including tissue, serum, and urine, and many metabolites have been described as promising biomarkers for HCC [20,21,22,23,24,25]. However, these studies have mainly focused on HCC caused by viral hepatitis, including hepatitis B and C, while no studies have specifically explored diagnostic biomarkers for T2DM(+) HCC.

In this study, we aimed to explore the dysregulated small molecule metabolites in T2DM(+) HCC compared to T2DM patients and to find potential biomarkers for the surveillance and early detection of HCC in the T2DM population. Serum metabolomic profiles of T2DM and T2DM(+) HCC patients in the discovery cohort were determined by untargeted metabolomic analysis, and the metabolites that were differentially expressed in T2DM(+) HCC compared with T2DM patients were identified. Subsequently, targeted metabolite analyses were used to validate the identified metabolites in the discovery cohort and another independent validation cohort. In addition, the diagnostic potential of these metabolites and their correlations with clinicopathologic variables of HCC patients were evaluated as well. Overall, our study is the first to identify novel metabolite biomarkers for T2DM(+) HCC by metabolomic analysis. 

## 2. Results

### 2.1. Serum Metabolomic Profiling Identified Significantly Dysregulated Metabolites in T2DM(+) HCC

**Figure 1:**
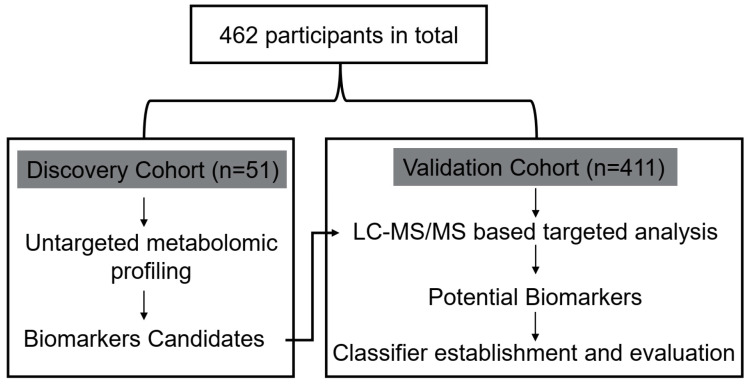
Design of the study.

**Figure 2:**
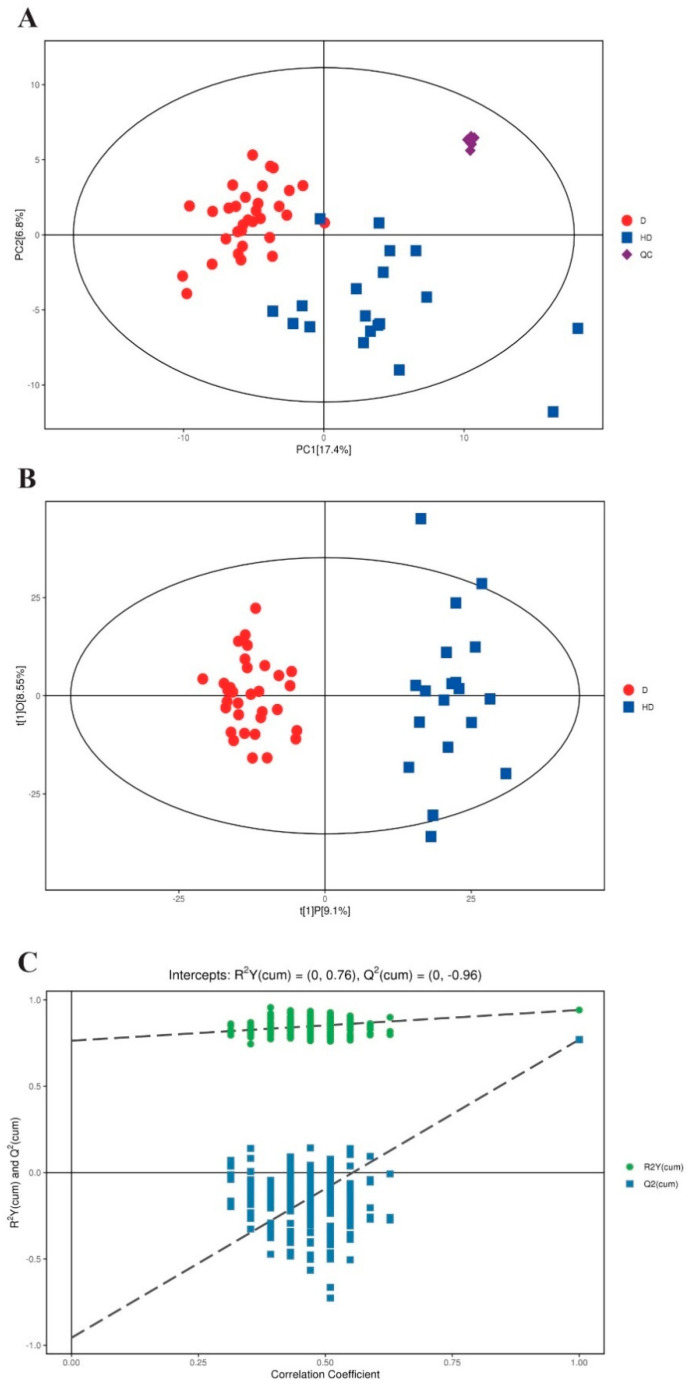
Metabolic profiling of serum samples from patients with T2DM and T2DM(+) HCC in the discovery cohort. (**A**) The PCA score plot based on the combinational data of ESI+ and ESI− modes. (**B**) The OPLS–DA score plot based on the combinational data of ESI+ and ESI− modes. (**C**) Validation of the OPLS–DA model by the permutation test. Colors and shapes display the subjects from different groups. D refers to T2DM patients, and HD refers to T2DM(+) HCC patients. T2DM, type 2 diabetes mellitus; HCC, hepatocellular cancer; PCA, principal component analysis; OPLS–DA, orthogonal projections to latent structures–discriminate analysis; QC, quality control.

The workflow scheme of the study is presented in Figure 1, and the demographic and clinical variables of the included participants are summarized in Table 1. Metabolomic profiling of serum samples from patients with T2DM and T2DM(+) HCC in the discovery cohort was carried out, and the pooled QC samples were evenly inserted into the running sequence to monitor analytical stability. Figure S1 presents the typical base peak chromatograms from ESI+ and ESI− mode. The QC samples were clustered tightly in the PCA score plot (Figure 2A), indicating that the stability and repeatability of the present analytical method was satisfactory. In addition, the consistent retention time and response intensity of the internal standard in the QC samples (Figure S2) further confirmed the reliability of the metabolomic analyses. Subsequently, the supervised OPLS-DA model revealed that a clear separation between the T2DM and T2DM(+) HCC groups. The cumulative R^2^Y and Q^2^ were 0.94 and 0.77, respectively, suggesting a high predictive ability of the model (Figure 2B). No overfitting was observed according to the results of the permutation test (the cumulative R^2^Y-intercept and Q^2^-intercept were 0.76 and −0.96, respectively) (Figure 2C). 

**Figure 3:**
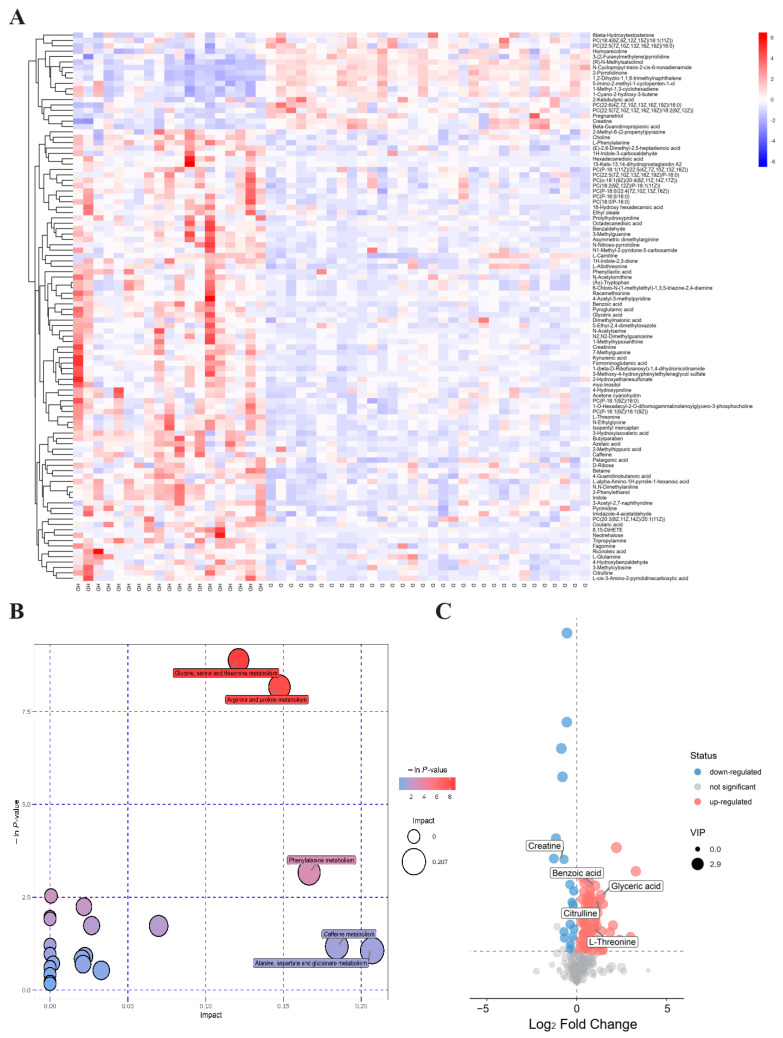
Dysregulated metabolites between T2DM and T2DM(+) HCC patients identified in metabolic profiling. (**A**) Hierarchical clustering showing the dysregulated metabolites between T2DM and T2DM(+) HCC patients. Each column represents a sample, and each row represents a metabolite. (**B**) Pathway enrichment analysis of dysregulated metabolites identified in the T2DM(+) HCC group versus the T2DM group. (**C**) The volcano plot depicts the difference of metabolites between T2DM and T2DM(+) HCC patients. A dot represents a metabolite. D refers to T2DM patients, and HD refers to T2DM(+) HCC patients. T2DM, type 2 diabetes mellitus; HCC, hepatocellular cancer.

In accordance with the conditions of VIP > 1 and *p* < 0.05, a total of 102 differential metabolites including 84 upregulated and 18 downregulated in T2DM(+) HCC patients were finally identified (Table S2). As shown in Figure 3A, the result of hierarchical clustering showed the distinguishable profiling of the differential metabolites between the T2DM and T2DM(+) HCC groups. In addition, pathway analysis was performed using the MetaboAnalyst database, and several amino acid metabolic pathways, including the glycine, serine and threonine metabolism, the arginine and proline metabolism, and the Phenylalanine metabolism, were revealed to be disturbed significantly in T2DM(+) HCC compared to T2DM patients (Figure 3B). The dysregulated metabolites in each pathway are displayed in Table 2. A volcano plot was constructed to visualize these metabolites, and the four metabolites most significantly upregulated and the one most significantly downregulated, including benzoic acid, glyceric acid, L-threonine, citrulline, and creatine (Figure 3C), were considered as candidate biomarkers for T2DM(+) HCC and subjected to the following targeted metabolite analyses. 

### 2.2. Validation of Differential Metabolites by Targeted Metabolite Analyses

**Figure 4:**
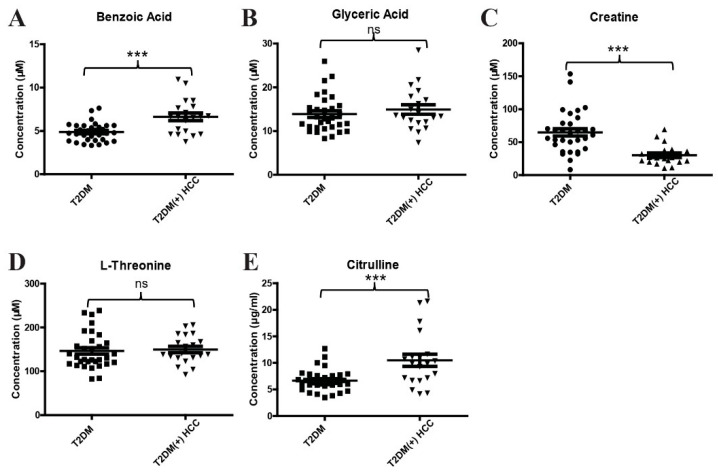
The expression profiles of several dysregulated metabolites in the discovery cohort. (**A**) The levels of serum benzoic acid in the discovery cohort. (**B**) The levels of serum glyceric acid in the discovery cohort. (**C**) The levels of serum creatine in the discovery cohort. (**D**) The levels of serum L-threonine in the discovery cohort. (**E**) The levels of serum citrulline in the discovery cohort. *** *p* value < 0.001; ns, not significant.

To validate the results of metabolomic profiling, a new method for simultaneous quantification of benzoic acid, glyceric acid, creatine, L-threonine, and citrulline by LC-MS/MS was developed. The methodology validation, including precision, linearity, linear range, and recovery, was performed, and the results suggest that this analytical method for targeted metabolites is reliable (Table S3). Consistently, the levels of benzoic acid (Figure 4A) and citrulline (Figure 4E) were significantly increased, and creatine (Figure 4C) was significantly decreased in T2DM(+) HCC patients compared to T2DM individuals in the discovery cohort. However, no significant difference was observed in the levels of glyceric acid (Figure 4B) and L-threonine (Figure 4D) between the two groups. 

**Figure 5:**
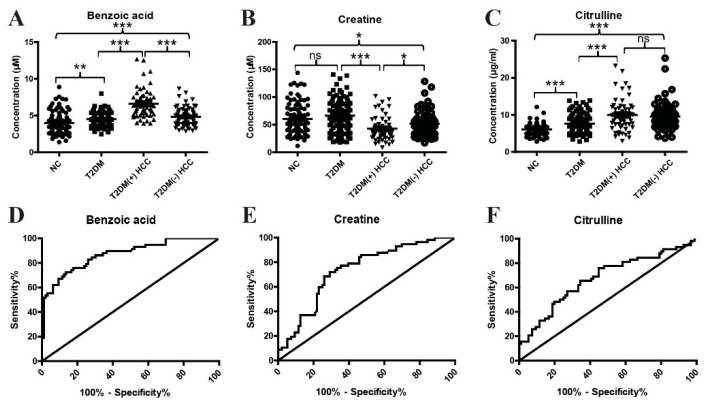
The diagnostic performance of benzoic acid, creatine, and citrulline in the validation cohort. (**A**) The levels of serum benzoic acid in NC, T2DM, T2DM(+) HCC, and T2DM(−) HCC individuals in the validation cohort. (**B**) The levels of serum creatine in NC, T2DM, T2DM(+) HCC, and T2DM(−) HCC individuals in the validation cohort. (**C**) The levels of serum citrulline in NC, T2DM, T2DM(+) HCC, and T2DM(−) HCC individuals in the validation cohort. (**D**) The ROC curve of benzoic acid for discriminating T2DM(+) HCC patients from T2DM patients. (**E**) The ROC curve of creatine for discriminating T2DM(+) HCC patients from T2DM patients. (**F**) The ROC curve of citrulline for discriminating T2DM(+) HCC patients from T2DM patients. NC, normal control; T2DM, type 2 diabetes mellitus; HCC, hepatocellular cancer; ROC, receiver operating characteristic. * *p* value < 0.05; ** *p* value < 0.01; *** *p* value < 0.001; ns, not significant.

Subsequently, to further confirm the above results, an independent validation cohort was included in the study. As shown in Figure 5A–C, the differential expressions of benzoic acid, creatine, and citrulline between T2DM and T2DM(+) HCC patients in the validation cohort was consistent with those in the discovery cohort. In addition, the levels of benzoic acid and creatine, but not citrulline, were also significantly different in T2DM(+) HCC and T2DM(−) HCC patients. We then evaluated the diagnostic potential of benzoic acid, creatine, and citrulline in T2DM(+) HCC using ROC curves. As shown in Figure 5D and Table 3, benzoic acid exhibited an AUC of 0.87 in discriminating T2DM(+) HCC patients from T2DM individuals, and the optimal sensitivity and specificity values were 72.41% and 86.46%, respectively. In addition, creatine exhibited an AUC of 0.73, and the optimal sensitivity and specificity values were 71.93% and 70.83%, respectively (Figure 5E and Table 3). Citrulline exhibited an AUC of 0.67, and the optimal sensitivity and specificity values were 65.52% and 65.63%, respectively (Figure 5F and Table 3). These results suggest that benzoic acid, creatine, and citrulline are dysregulated in T2DM(+) HCC and have diagnostic potential for the detection of T2DM(+) HCC in a T2DM population.

### 2.3. Evaluation of the Diagnostic Performance of the Metabolite Classifier That Incorporates Benzoic Acid, Creatine, and Citrulline

**Figure 6:**
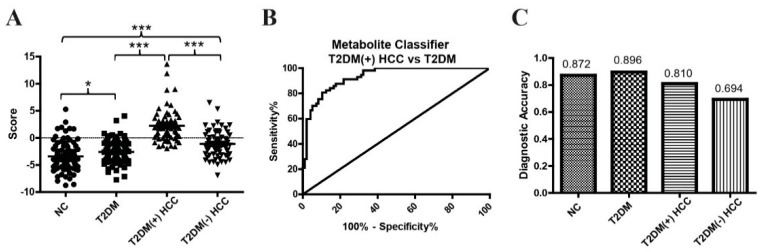
The diagnostic performance of the metabolite classifier in the validation cohort. (**A**) The scores of the metabolite classifier in NC, T2DM, T2DM(+) HCC, and T2DM(−) HCC individuals in the validation cohort. (**B**) The ROC curve of the metabolite classifier including benzoic acid, creatine, and citrulline for discriminating T2DM(+) HCC patients from T2DM patients. (**C**) The diagnostic accuracy of the metabolite classifier for the diagnosis of NC, T2DM, T2DM(+) HCC, and T2DM(−) HCC individuals. NC, normal control; T2DM, type 2 diabetes mellitus; HCC, hepatocellular cancer; ROC, receiver operating characteristic. * *p* value < 0.05; *** *p* value < 0.001.

Next, we determined whether the combination of benzoic acid, creatine, and citrulline could improve the diagnostic efficacy for T2DM(+) HCC. Logistic regression based on benzoic acid, creatine, and citrulline was used to construct a metabolite classifier. This metabolite classifier for the detection of T2DM(+) HCC was constructed as follows: the classifier score = 1.567 × [benzoic acid] − 0.044× [creatine] + 0.1864 × [citrulline] − 8.1713. As shown in Figure 6A, the classifier score was significantly higher in T2DM(+) HCC patients compared to T2DM and T2DM(−) HCC patients. The metabolite classifier exhibited better diagnostic performance in differentiating T2DM(+) HCC patients from T2DM individuals (at the optimal cut-off point, AUC = 0.93, sensitivity = 80.70%, and specificity = 89.58%) than any single metabolite (Figure 6B and Table 4). The diagnostic accuracy of the metabolite classifier was 0.872 for NC, 0.896 for T2DM, 0.810 for T2DM(+) HCC, and 0.694 for T2DM(−) HCC patients (Figure 6C). The correlations between the metabolite classifier and the clinical characteristics of T2DM(+)HCC individuals are listed in Table 5. 

**Figure 7:**
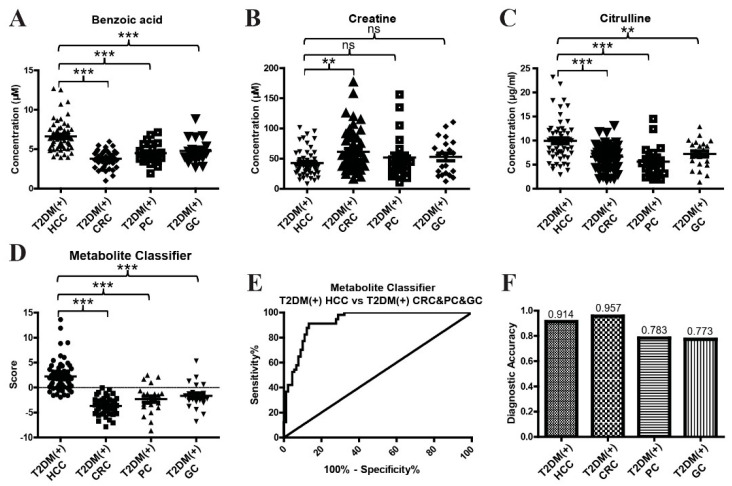
The specificity of the metabolite classifier in the diagnosis of T2DM(+) HCC patients. (**A**) The levels of serum benzoic acid in T2DM(+) HCC, T2DM(+) CRC, T2DM(+) PC, and T2DM(+) GC patients in the validation cohort. (**B**) The levels of serum creatine in T2DM(+) HCC, T2DM(+) CRC, T2DM(+) PC, and T2DM(+) GC patients in the validation cohort. (**C**) The levels of serum citrulline in T2DM(+) HCC, T2DM(+) CRC, T2DM(+) PC, and T2DM(+) GC patients in the validation cohort. (**D**) The scores of the metabolite classifier in T2DM(+) HCC, T2DM(+) CRC, T2DM(+) PC, and T2DM(+) GC patients in the validation cohort. (**E**) The ROC curve of the metabolite classifier including benzoic acid, creatine, and citrulline for discriminating T2DM(+) HCC patients from T2DM(+) CRC&PC&GC patients. (**F**) The diagnostic accuracy of the metabolite classifier for the diagnosis of T2DM(+) HCC, T2DM(+) CRC, T2DM(+) PC, and T2DM(+) GC patients. T2DM, type 2 diabetes mellitus; HCC, hepatocellular cancer; CRC, colorectal cancer; PC, pancreatic cancer; GC, gastric cancer; ROC, receiver operating characteristic. ** *p* value < 0.01; *** *p* value < 0.001; ns, not significant.

As T2DM was closely associated with the occurrence of CRC, PC, GC, and HCC [26,27,28], T2DM(+) CRC, T2DM(+) PC, and T2DM(+) GC patients were included in the validation cohort to assess the specificity of the metabolite classifier for T2DM(+) HCC. At first, we compared the levels of benzoic acid, creatine, and citrulline in T2DM(+) HCC, T2DM(+) CRC, T2DM(+) PC, and T2DM(+) GC patients. Interestingly, the levels of benzoic acid (Figure 7A) and citrulline (Figure 7C) in T2DM(+) CRC, T2DM(+) PC, and T2DM(+) GC patients were significantly lower than those in T2DM(+) HCC patients. However, although the level of creatine in T2DM(+) HCC patients was significantly lower than that in T2DM(+) CRC patients, there was no significant difference between T2DM(+) HCC patients and T2DM(+) PC and T2DM(+) GC patients (Figure 7B). The classifier score was calculated, and T2DM(+) HCC patients showed significantly higher scores than T2DM(+) CRC, T2DM(+) PC, and T2DM(+) GC patients (Figure 7D). We then evaluated the diagnostic performance of the metabolite classifier to distinguish between T2DM(+) HCC patients and T2DM(+) CRC, T2DM(+) PC, and T2DM(+) GC patients. As shown in Figure 7E and Table 4, the metabolite classifier exhibited an AUC of 0.93, and the optimal sensitivity and specificity were 91.23% and 86.67%, respectively. The diagnostic accuracy of the metabolite classifier was 0.914 for T2DM(+) HCC, 0.957 for T2DM(+) CRC, 0.783 for T2DM(+) PC, and 0.773 for T2DM(+) GC patients (Figure 7F), suggesting the specificity of the metabolite classifier for the detection of T2DM(+) HCC. These data clearly suggest that the metabolite classifier that incorporates benzoic acid, creatine, and citrulline showed high diagnostic efficacy in detecting T2DM(+) HCC from T2DM and other T2DM(+) tumors, including T2DM(+) CRC, T2DM(+) PC, and T2DM(+) GC. 

### 2.4. The Diagnostic Performance of the Metabolite Classifier in Small-Size, Early-Stage, and AFP-Negative T2DM(+) HCC 

**Figure 8:**
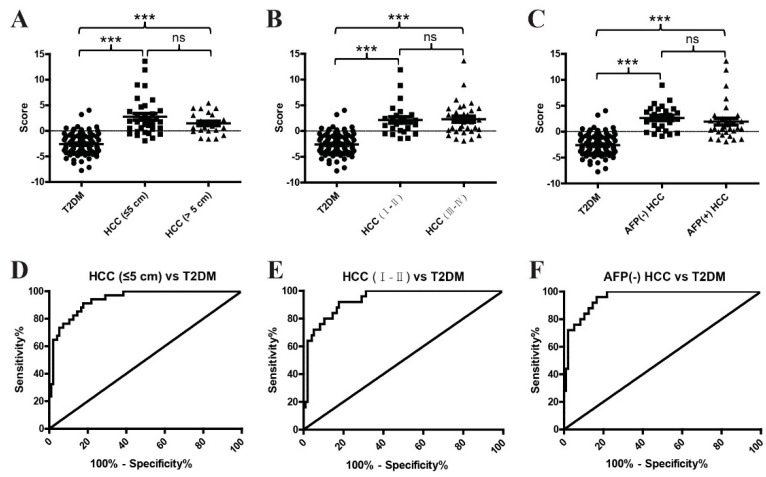
The role of the metabolite classifier in the diagnosis of small–size, early–stage, and AFP(−) T2DM(+) HCC patients. (**A**) The scores of the metabolite classifier in T2DM(+) HCC patients with small (size ≤ 5) and large (size > 5) tumors compared with T2DM patients. (**B**) The scores of the metabolite classifier in T2DM(+) HCC patients with early-stage (stage I–II) and late–stage (stage III–IV) tumors compared with T2DM patients. (**C**) The scores of the metabolite classifier in T2DM(+) HCC patients with AFP(−) and AFP (+) tumors compared with T2DM patients. (**D**) The ROC curve of the metabolite classifier for discriminating T2DM(+) HCC patients with small–size tumors from T2DM patients. (**E**) The ROC curve of the metabolite classifier for discriminating T2DM(+) HCC patients with early–stage tumors from T2DM patients. (**F**) The ROC curve of the metabolite classifier for discriminating T2DM(+) HCC patients with AFP(−) tumors from T2DM patients. AFP, alpha-fetoprotein; T2DM, type 2 diabetes mellitus; HCC, hepatocellular cancer; ROC, receiver operating characteristic. *** *p* value < 0.001; ns, not significant.

As small-size, early-stage, and AFP-negative [AFP(−)] HCC cases are usually difficult to detect, we assessed the diagnostic value of the metabolite classifier in these tumors. As shown in Figure 8A,D and Table 6, the metabolite classifier exhibited an AUC of 0.94 in discriminating small-size T2DM(+) HCC patients from T2DM patients, and the optimal sensitivity and specificity values were 91.18% and 82.29%, respectively. Similarly, the metabolite classifier exhibited an AUC of 0.94 in discriminating early-stage (stage Ⅰ–Ⅱ) T2DM(+) HCC patients from T2DM patients, and the optimal sensitivity and specificity values were 92.00% and 82.29%, respectively (Figure 8B,E and Table 6). In addition, the metabolite classifier also showed great diagnostic performance with an AUC of 0.96 in differentiating AFP(−) T2DM(+) HCC patients from T2DM individuals, and the corresponding sensitivity and specificity values were 96% and 83.33%, respectively (Figure 8C,F and Table 6). Collectively, these results suggest the outstanding performance of our metabolite classifier in diagnosing small-size, early-stage, and AFP(−) T2DM(+) HCC, implying the critical value of this classifier in the early diagnosis of T2DM(+) HCC. 

### 2.5. The Combination of the Metabolite Classifier and AFP in the Diagnosis of T2DM(+) HCC

**Figure 9:**
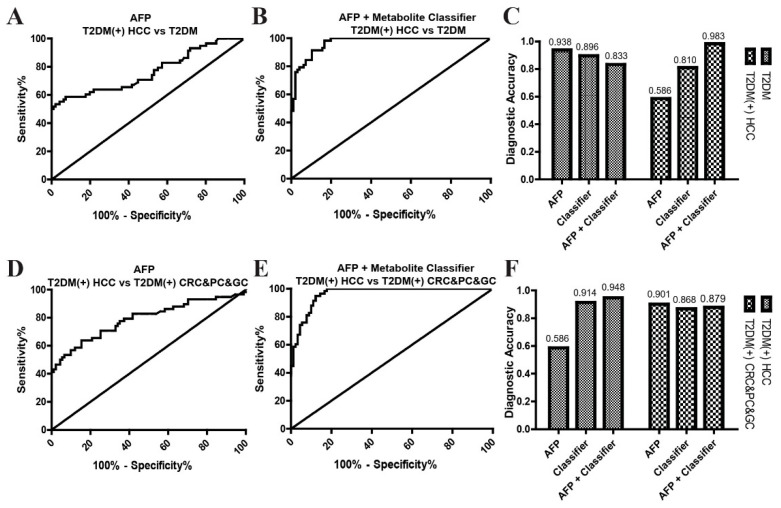
The combination of AFP and the metabolite classifier for T2DM(+) HCC diagnosis. (**A**) ROC curve of AFP for discriminating T2DM(+) HCC patients from T2DM patients. (**B**) ROC curve of the combination of AFP and the metabolite classifier for discriminating T2DM(+) HCC patients from T2DM patients. (**C**) The corresponding diagnostic accuracy for the diagnosis of T2DM and T2DM(+) HCC patients. (**D**) ROC curve of AFP for discriminating T2DM(+) HCC patients from T2DM(+) CRC&PC&GC patients. (**E**) ROC curve of the combination of AFP and the metabolite classifier for discriminating T2DM(+) HCC patients from T2DM(+) CRC&PC&GC patients. (**F**) The corresponding diagnostic accuracy for the diagnosis of T2DM(+) HCC and T2DM(+) CRC&PC&GC individuals. AFP, alpha-fetoprotein; T2DM, type 2 diabetes mellitus; HCC, hepatocellular cancer; CRC, colorectal cancer; PC, pancreatic cancer; GC, gastric cancer; ROC, receiver operating characteristic.

Finally, we determined whether the combination of the metabolite classifier and AFP could further improve the diagnostic accuracy for T2DM(+) HCC. As shown in Figure 9 and Table 7, the combination of the metabolite classifier and AFP exhibited much better diagnostic performance than AFP alone in differentiating, from T2DM patients, T2DM(+) HCC patients (AUC: 0.97 vs. 0.76) or T2DM(+) CRC&PC&GC patients (AUC: 0.96 vs. 0.79). Although the diagnostic accuracy of the combination of the metabolite classifier and AFP was slightly lower than that of AFP alone for T2DM and T2DM(+) CRC&PC&GC patients, the combined panel showed much higher diagnostic accuracy than AFP alone for T2DM(+) HCC (Figure 9C,F and Table 7). In addition, the diagnostic performances of the combination of the metabolite classifier and AFP were better than those of the metabolite classifier alone, and the combination of the metabolite classifier and AFP offered a slightly higher diagnostic accuracy than the metabolite classifier alone for T2DM(+) HCC (Figure 6, Figure 7, and Figure 9 and Table 4 and Table 7). These results indicate that the combination of the metabolite classifier and AFP further improves the diagnostic accuracy for T2DM(+) HCC and has critical clinical significance in the surveillance and early detection of HCC in T2DM individuals. 

## 3. Discussion

In the present study, serum benzoic acid, creatine, and citrulline were found to be significantly dysregulated in T2DM(+) HCC patients compared to T2DM individuals. A metabolite classifier based on the three metabolites showed good diagnostic performance in differentiating T2DM(+) HCC patients from T2DM patients and patients with other T2DM(+) tumors, including CRC, PC, and GC. In addition, the metabolite classifier also offered high diagnostic accuracy in the detection of small-size, early-stage, and AFP(−) T2DM(+) HCC, and the combination of the metabolite classifier and AFP exhibited better diagnostic efficacy. 

The exploration of potential biomarkers for HCC surveillance in the T2DM population has become increasingly important due to the growing burden of T2DM worldwide and its close relationship with HCC [14,29]. There is no effective diagnostic biomarker for T2DM(+) HCC in clinical practice at present, and the screening of HCC tumors, especially those at early stages and small in size, from T2DM patients remains a challenge. In the present study, the serum metabolite classifier including benzoic acid, creatine, and citrulline was shown to effectively distinguish between T2DM and T2DM(+) HCC individuals, and it was capable of detecting small, early-stage, and AFP(−) T2DM(+) HCC with high sensitivity and specificity. Our study highlights the potential of this metabolite classifier in the early diagnosis of T2DM(+) HCC, which will help avoid delays in clinical treatment and have important implications for improving the prognosis of T2DM(+) HCC patients. 

An increasing body of research has suggested the potential of serum metabolites as diagnostic biomarkers for HCC. For example, it has been reported by a European group that many serum metabolites, such as phosphatidylcholines, sphingomyelins, triglycerides, amino acids, and cholesteryl esters, were altered in HCC, and some of them showed higher diagnostic sensitivity and specificity than AFP [30]. In addition, a Chinese group found that a serum metabolite panel including phenylalanyl-tryptophan and glycocholate, as well as the combination of betaine and propionylcarnitine, separated HCC from high-risk chronic hepatitis B and cirrhosis individuals with high performance [20,31]. Moreover, a two-metabolite panel including retinol and retinal and a four-metabolite panel including chenodeoxycholic acid, lysophosphatidylcholine (20:5), succinyladenosine, and uridine were also described to have considerable diagnostic values for HCC [23,32]. The results of these metabolomic analyses were inconsistent, possibly due to differences in the ethnicity and clinical characteristics of the patients included in these studies. In this study, all included HCC patients were divided into T2DM(+) and T2DM(−) groups, with the aim of exploring T2DM(+) HCC-specific diagnostic biomarkers. Finally, a metabolite classifier with high sensitivity and specificity was constructed for the surveillance and early detection of HCC in the T2DM population. 

Although the epidemiological studies have revealed a tight association between T2DM and HCC, the pathophysiological mechanism by which T2DM promoted the development of HCC remains intriguing. It has been demonstrated that insulin resistance, one of the major hallmarks of T2DM, can lead to the production of proinflammatory mediators, oxidative stress, and alterations in gut microbiota, which contribute significantly to hepatic carcinogenesis [33]. In addition, insulin-like growth factor (IGF), AMP-activated protein kinase (AMPK), vascular endothelial growth factor (VEGF), and some other pathways are involved in the initiation and progression of diabetes-associated HCC [29]. Several studies have also indicated that small molecule metabolites were critical for T2DM(+) HCC tumorigenesis. For example, metabolomic profiling of T2DM(+) and T2DM(−) HCC tissues suggested that some metabolites, including 2-hydroxystearate, were only upregulated in T2DM(+) HCC tissues and might play an important role in the development of HCC in T2DM patients [34]. In addition, 8-hydroxydeoxyguanosine, L-arginine, and glucose metabolites were reported to be overexpressed in T2DM and non-alcoholic steatohepatitis (NASH)-associated HCC and might participate in cell proliferation, mTOR pathways, and the activation of oxidative stress resistance [35]. However, the metabolic abnormalities in T2DM(+) HCC patients compared to T2DM patients are still largely unclear at present. In this study, the serum metabolomic profiles in T2DM and T2DM(+) HCC patients were compared, and some amino acids and their derivatives were revealed to be dysregulated significantly in T2DM(+) HCC patients, indicating that amino acid metabolism might influence the development of HCC in T2DM patients. 

Amino acid metabolism is usually aberrantly modulated in cancer and has diverse and critical roles in various cancers [36,37]. However, their dysregulation in HCC remains controversial. For example, several studies have indicated that creatine was significantly reduced in the serum and urine samples of HCC patients [38,39,40], while other studies have reported that it was increased in HCC patients compared to controls [41,42]. Phenylalanine, the central metabolite in the phenylalanine metabolism pathway, was frequently found to be significantly altered in HCC, but the trend of its alteration in different studies was inconsistent as well [24,43,44,45,46]. These inconsistencies might be due to differences in the HCC cases included in these studies. In the present study, T2DM(+) HCC patients were recruited specifically to explore the metabolite characteristics in this type of HCC. Consistent with some previous studies, the T2DM(+) HCC group showed significantly decreased creatine and increased phenylalanine compared to the T2DM group. In addition, benzoic acid, another metabolite in the phenylalanine metabolism pathway, was also revealed to be upregulated in HCC, which has rarely been reported in previous studies. Our results further support that the amino acid metabolism is disturbed in HCC cases, and amino acids and their derivatives might serve as diagnostic biomarkers for HCC.

## 4. Conclusions

The present study is the first to report that serum benzoic acid, creatine, and citrulline are increased in T2DM(+) HCC patients, and the metabolite classifier that incorporates benzoic acid, creatine, and citrulline exhibits a satisfactory diagnostic performance for T2DM(+) HCC. Therefore, the metabolite classifier holds great promise for clinical application in the surveillance and early detection of HCC in T2DM individuals. In the future, multicenter prospective studies are needed to further validate the clinical value of this classifier. 

## 5. Materials and Methods

### 5.1. Study Population

In the present study, a total of 462 participants, including patients with HCC, colorectal cancer (CRC), gastric cancer (GC), pancreatic cancer (PC), and T2DM as well as normal controls (NC) were recruited in Peking University People’s Hospital. In the discovery stage, 32 T2DM patients and 19 T2DM(+) HCC patients were included and subjected to untargeted metabolomic analyses. In the validation stage, 94 NCs, 96 T2DM patients, 58 T2DM(+) HCC patients, 72 T2DM-negative [T2DM(−)] HCC patients, 46 T2DM(+) CRC patients, 22 T2DM(+) GC patients, and 23 T2DM(+) PC patients were included and subjected to targeted metabolite analyses. This study was approved by the Ethical Review Committee of Peking University People’s Hospital and performed according to the principles of the Declaration of Helsinki. 

The diagnostic criteria for T2DM [47] were typical symptoms of diabetes (polydipsia, polyuria, polyphagia, and weight loss) plus at least one of the following: (1) random blood glucose ≥ 11.1 mmol/L; (2) fasting blood glucose (FBG) ≥ 7.0 mmol/L; (3) 2 h blood glucose ≥ 11.1 mmol/L during an oral glucose tolerance test (OGTT); (4) hemoglobin A1c (HbA1c) ≥ 6.5%. The diagnosis of HCC, CRC, GC, and PC were confirmed by histopathology, and patients with a history of other malignancies were excluded. In addition, a subset of HCC patients and all CRC, GC, and PC patients included in this study had T2DM. The enrolled NC subjects were healthy individuals who had received physical examinations in our hospital. Peripheral blood samples were collected from the NC and T2DM groups during regular examination and from the HCC, CRC, GC, and PC patients before surgery under fasting conditions. 

### 5.2. Chemicals and Reagents

Acetonitrile (gradient grade for liquid chromatography) was obtained from Fisher Chemical. Methanol (gradient grade for liquid chromatography), ammonia hydroxide, formic acid, and citrulline were purchased from Sigma-Aldrich. Ammonium acetate was obtained from Aladdin. Distilled water was purchased from Watsons. Glyceric acid, benzoic acid, L-threonine, and creatine were obtained from Tokyo Chemical Industry. The isotope-labeled internal standard mixture for metabolomic analyses was from Biotree Biomedical Technology. The citrulline analogue L-glutamic acid -2,3,3,4,4-d5 was purchased from Sigma-Aldrich as well and used as an internal standard for targeted metabolite analyses. 

### 5.3. Measurement of Clinical Indicators

The peripheral blood sample was collected and subjected to centrifugation (4000 rpm for 10 min) to separate serum for each individual. Serum FBG was measured by an AU5832 automatic biochemical analyzer (Beckman Coulter, Brea, CA, USA). Serum HBV surface antigen (HBsAg) and HCV antibody were detected by the automatic chemiluminescent microparticle immunoassay analyzer ARCHITECT i2000 SR (Abbott Laboratories, Abbott Park, IL, USA). Serum AFP was determined by the automatic electrochemiluminescence immunoassay analyzer Cobas e801 (Roche Diagnostics, Penzberg, Germany). All measurements were carried out with the original manufacturers’ reagents according to the manufacturers’ standard operating procedures. 

### 5.4. Untargeted Metabolomic Analyses

The pretreatment of serum specimens was as follows. At first, 100 μL of serum were added to a 400 μL extract solution (acetonitrile: methanol = 1:1, containing isotope labeled internal standard mixture) and mixed by vortexing. The sample was then sonicated for 10 min in an ice-water bath and incubated for 1 h at −40 °C to precipitate proteins. Subsequently, the sample was centrifuged at 12,000 rpm for 15 min at 4 °C, and the supernatant was transferred to a fresh glass vial for metabolomic analysis. The quality control (QC) sample was prepared by mixing the supernatants of all samples in equal amounts. 

Metabolomic analyses were performed on an UHPLC/Q Exactive HFX mass spectrometer (Thermo Fisher Scientific, Cleveland, OH, USA) using an ACQUITY UPLC BEH Amide column (2.1 mm × 100 mm, 1.7 μm, Waters, Milford, MA, USA). Mobile Phase A was H_2_O containing 25 mmol/L ammonium acetate and 25 mmol/L ammonia hydroxide (pH = 9.75), and Mobile Phase B was acetonitrile. The auto-sampler temperature was set at 4 °C, and the column oven was maintained at 30 °C. The injection volume was 2 μL, and the sample sequence was random. The flow rate was 0.5 mL/min. The elution gradient program was as follows: 0–0.5 min, 95% B; 0.5–7 min, 95–65% B; 7–8 min, 65–40% B; 8–9 min, 40% B; 9–9.1 min, 40–95% B; 9.1–12 min, 95% B. The ion spray voltages were 3600 V in the ESI+ mode and −3200 V in the ESI− mode. The capillary temperature was maintained at 350 °C with the sheath gas at 30 arbitrary units and the aux gas at 25 arbitrary units. The scan range of the mass spectrum was set from 70 to 1050 m/z. The MS resolution was set to 120,000. The collision energy was set to 10, 30, or 60 eV in the ESI+ mode, and −10, −30, or −60 eV in the ESI− mode. The MS/MS spectra were acquired by the acquisition software Xcalibur (Thermo Fisher Scientific) on information-dependent acquisition (IDA) mode. 

### 5.5. Targeted Metabolite Analyses

Calibration standard mixtures were prepared according to the following procedures. At first, twenty serum samples were mixed in equal amounts. Seven standard mixtures of benzoic acid, glyceric acid, creatine, L-threonine, and citrulline were then prepared by 10-fold serial dilutions using the mixed serum. The concentrations of the standard mixtures were determined by the concentration ranges of these metabolites in serum as described in previous studies [48,49,50,51,52]. In addition, two QC samples (low-level and high-level) were prepared by spiking appropriate concentrations of benzoic acid, glyceric acid, creatine, L-threonine, and citrulline into the mixed serum. 

Subsequently, the pretreatment of calibration standard mixtures, QC, and serum samples was carried out according to the same procedure as follows: A 100 μL sample was mixed with a 200 μL extract solution (acetonitrile: methanol = 1:1, containing isotope-labeled internal standard L-glutamic acid -2,3,3,4,4-d5) by vortexing, and centrifuged at 12,000 rpm for 15 min at 4 °C. Afterward, 200 μL of the supernatant was then transferred to a fresh tube, and 800 μL of distilled water were added to dilute the supernatant. The diluted sample could be directly used for quantitative liquid chromatography tandem mass spectrometry (LC-MS/MS) analysis. 

Targeted metabolite analyses were performed on a Jasper HPLC system coupled to a Triple Quadrupole 4500MD mass spectrometer system (SCIEX, Framingham, MA, USA). A Kinetex F5 column (3 mm × 100 mm, 2.6 μm, Phenomenex, Torrance, CA, USA) was used to analyze the pretreated samples. Mobile Phase A consisted of 10 mmol/L ammonium acetate and 0.1% formic acid in H_2_O, and Mobile Phase B consisted of 2.5 mmol/L ammonium acetate and 0.1% formic acid in 95% acetonitrile. The auto-sampler temperature was set at 10 °C, and the column oven was maintained at 45 °C. The injection volume was 5 μL, and the flow rate was 0.4 mL/min. The elution gradient program was as follows: 0–1 min, 1% B; 1–2.5 min, 1–95% B; 2.5–3.9 min, 95% B; 3.9–4.0 min, 95–1% B; 4–5 min, 1% B. The MS detection was conducted with a Turbo Spray probe in positive ion mode, except for the analysis of glyceric acid and benzoic acid. The metabolites were tuned individually, and the optimal multiple reaction monitoring (MRM) transitions were established (Table S1). The ion spray voltage was set at 4500 V in the ESI+ mode and −4500 V in the ESI− mode. The capillary temperature was maintained at 400 °C with the curtain gas at 25 arbitrary units and the collision gas at 9 arbitrary units. The MS/MS spectra were acquired by the acquisition software Analyst MD 1.6.3 (SCIEX) and quantified by MultiQuant MD 3.0.3 software (SCIEX). 

### 5.6. Statistical Analysis 

The processed metabolomic data were subjected to multivariate analysis using SIMCA 16.0.2 software package (Sartorius Stedim Data Analytics AB, Umea, Sweden). An unsupervised principal component analysis (PCA) with unit variance scaling was performed to reflect the distribution of the samples from different groups and monitor the stability of the metabolomic analyses. A supervised model of orthogonal projections to latent structures-discriminate analysis (OPLS-DA) with unit variance scaling was conducted to maximize the distance between groups and identify variables with important contributions to classification according to the scores of variable importance in the projection (VIP). A permutation test was performed 200 times to determine the risk of overfitting. The metabolites with VIP > 1 and *p* < 0.05 (Student’s *t* test) were considered as significantly dysregulated metabolites. Hierarchical clustering analysis was performed, and a volcano plot was constructed to represent the dysregulated metabolites between groups. The MetaboAnalyst database (http://www.metaboanalyst.ca/, accessed on 5 February 2022) was applied to reveal the significantly disturbed metabolic pathways in T2DM(+) HCC patients compared to the T2DM group. 

For targeted metabolite analysis, data were analyzed using GraphPad Prime 5.01 (GraphPad Software, San Diego, CA, USA), SPSS 20.0 software (IBM, Poughkeepsie, NY, USA), or MedCalc 20.027 (MedCalc Software, Mariakerke, Belgium). All continuous variables were expressed as mean ± standard deviation (SD). The variables following a Gaussian distribution were compared using a Student’s *t* test, and those that did not follow a Gaussian distribution were compared using a Mann–Whitney U test. Binary logistic regression was used to build the classifier based on the dysregulated metabolites. Receiver operating characteristic (ROC) curves were applied to evaluate the diagnostic performances. The cut-off points with optimal sensitivity and specificity values were determined by Youden’s index. The correlations between variables were evaluated by the chi-square test. The *p* value < 0.05 was considered statistically significant. 

## Figures and Tables

**Table 1:** The demographic and clinical variables of individuals included in this study.

	Discovery Cohort		Validation Cohort						
**Variables**	T2DM	T2DM(+) HCC	NC	T2DM	T2DM(+) HCC	T2DM(−) HCC	T2DM(+) CRC	T2DM(+) PC	T2DM(+) GC
	N = 32	N = 19	N = 94	N = 96	N = 58	N = 72	N = 46	N = 23	N = 22
**Age**	56.47 ± 11.37	64.32 ± 9.21	43.56 ± 15.02	55.15 ± 12.92	61.48 ± 9.70	56.11 ± 12.97	68.20 ± 10.99	69.04 ± 7.99	67.50 ± 11.49
**Gender Male/Female**	20/12	17/2	32/62	65/31	49/9	57/15	28/18	16/7	15/7
**FBG** **(mmol/L)**	7.43 ± 1.43	9.75 ± 4.94	4.99 ± 0.42	8.17 ± 2.37	8.60 ± 3.69	5.10 ± 0.55	7.72 ± 2.02	7.88 ± 2.41	8.80 ± 4.23
**AFP** **>7/** **≤** **7 ng/mL**	3/29	11/8	2/92	6/90	34/24	43/29	2/44	3/20	4/18

Abbreviations: T2DM, type 2 diabetes mellitus; HCC, hepatocellular cancer; NC, normal control; CRC, colorectal cancer; PC, pancreatic cancer; GC, gastric cancer; FBG, fasting blood glucose; AFP, alpha-fetoprotein.

**Table 2:** The dysregulated metabolites in several amino acid metabolic pathways.

Pathway	Total	Hits	*p* Value	Impact	Metabolite	MS2 Score	VIP	*p* Value	FC	Log_FC
**Glycine, Serine and Threonine Metabolism**	48	6	0.0001	0.1212	Choline	0.9998	1.8147	0.0003	1.2297	0.2983
					Glyceric acid	0.9378	2.4864	0.0002	2.5316	1.3401
					Betaine	0.9994	1.7816	0.0027	1.2823	0.3588
					L-Threonine	0.8118	1.2569	0.0051	1.8870	0.9161
					Creatine	0.9999	1.6842	5.52 × 10^−6^	0.6108	−0.7112
					L-Allothreonine	0.8758	1.4867	0.0083	1.3063	0.3855
**Arginine and Proline Metabolism**	77	7	0.0003	0.1474	L-Glutamine	0.6030	1.1105	0.0370	1.3353	0.4172
					Citrulline	0.9887	2.3293	0.0003	2.4283	1.2800
					N-Acetylornithine	0.7652	1.1535	0.0307	1.5949	0.6735
					Hydroxyproline	0.9951	1.1415	0.0350	1.5815	0.6613
					Creatine	0.9999	1.6842	5.52 × 10^−6^	0.6108	−0.7112
					Creatinine	0.9998	1.7932	0.0084	1.3825	0.4672
					4-Guanidinobutanoic acid	0.9866	1.8229	0.0001	1.3149	0.3950
**Phenylalanine Metabolism**	45	3	0.0422	0.1665	L-Phenylalanine	0.9937	2.2553	0.0001	1.3532	0.4363
					Phenylethyl alcohol	0.9373	2.1139	0.0001	1.6448	0.7179
					Benzoic acid	0.9950	2.6529	0.0001	2.0267	1.0192

Abbreviations: VIP, variable importance in the projection; FC, fold change.

**Table 3:** The diagnostic performance of benzoic acid, creatine, and citrulline for the detection of T2DM(+) HCC in a T2DM population.

	AUC (95%CI)	Sensitivity (%)	Specificity (%)	*p* Value
Benzoic acid	0.87 (0.81–0.93)	72.41	86.46	<0.0001
Creatine	0.73 (0.65–0.81)	71.93	70.83	<0.0001
Citrulline	0.67 (0.58–0.76)	65.52	65.63	0.0003

**Table 4:** The diagnostic performance of the metabolite classifier for the detection of T2DM(+) HCC.

Groups	AUC (95%CI)	Sensitivity (%)	Specificity (%)	*p* Value
**T2DM(+) HCC vs. T2DM**	0.93 (0.89–0.97)	80.70	89.58	<0.0001
**T2DM(+) HCC vs. T2DM(+) CRC&PC&GC**	0.93 (0.89–0.97)	91.23	86.67	<0.0001

**Table 5:** Correlation of the classifier score with clinical characteristics in T2DM(+) HCC patients in the validation cohort.

Variables	N	Classifier Score		*p* Value
		Low (n = 29)	High (n = 29)	
**Age**				0.79
≤60 y	29	14	15	
>60 y	29	15	14	
**Gender**				0.28
Male	49	26	23	
Female	9	3	6	
**BMI**				0.57
≥24 kg/m^2^	41	22	19	
<24 kg/m^2^	17	7	10	
**FBG (mmol/L)**				0.55
		8.91 ± 3.57	8.31 ± 3.84	
**AFP**				0.06
>7 ng/mL	33	20	13	
≤7 ng/mL	25	9	16	
**HBV**				0.10
Positive	38	22	16	
Negative	20	7	13	
**HCV**				0.15
Positive	2	2	0	
Negative	56	27	29	
**Cirrhosis**				0.13
Yes	43	24	19	
No	15	5	10	
**Alcohol Consumption**				0.75
Yes	13	6	7	
No	45	23	22	
**Tumor Size**				0.79
>5 cm	23	12	11	
≤5 cm	35	17	18	
**Tumor Number**				0.57
=1	40	19	21	
>1	18	10	8	
**CNLC Stage**				0.79
Ⅰ–Ⅱ	25	13	12	
Ⅲ–Ⅳ	33	16	17	
**Vascular invasion**				0.79
Yes	23	11	12	
No	35	18	17	

Abbreviations: FBG, fasting blood glucose; AFP, alpha-fetoprotein; HBV, hepatitis B virus; HCV, hepatitis C virus; CNLC, China liver cancer staging.

**Table 6:** The diagnostic performance of the metabolite classifier for the detection of small-size, early-stage, and AFP(−) T2DM(+) HCC.

Groups	AUC (95%CI)	Sensitivity (%)	Specificity (%)	*p* Value
HCC (≤5 cm) vs. T2DM	0.94 (0.90–0.98)	91.18	82.29	<0.0001
HCC (Ⅰ–Ⅱ) vs. T2DM	0.94 (0.89–0.98)	92	82.29	<0.0001
AFP(−) HCC vs. T2DM	0.96 (0.92–0.99)	96	83.33	<0.0001

**Table 7:** The combination of the metabolite classifier and AFP for T2DM(+) HCC detection.

Groups	AUC (95%CI)	Sensitivity (%)	Specificity (%)	*p* Value
**AFP**				
T2DM(+) HCC vs. T2DM	0.76 (0.68–0.85)	56.90	92.91	<0.0001
T2DM(+) HCC vs. T2DM(+) CRC&PC&GC	0.79 (0.71–0.87)	56.90	89.01	<0.0001
**AFP + Classifier**				
T2DM(+) HCC vs. T2DM	0.97 (0.95–0.99)	98.28	83.33	<0.0001
T2DM(+) HCC vs. T2DM(+) CRC&PC&GC	0.96 (0.94–0.99)	94.83	87.78	<0.0001

## Data Availability

The original contributions presented in the study are all included in the article/Supplementary Material.

## Supplementary Materials

The following supporting information can be downloaded at https://www.mdpi.com/article/10.3390/metabo12070610/s1. Figure S1: The representative base peak chromatograms under the ESI+ (A) and ESI− (B) mode. Figure S2: The retention time and response intensity of the internal standard in the QC samples under the ESI+ (A) and ESI− (B) mode in the metabolomic analyses. Table S1: MRM settings for benzoic acid, glyceric acid, creatine, L-threonine, and citrulline. Table S2: List of differential metabolites identified in the metabolomic analyses. Table S3: The results of methodology validation for the quantification of serum benzoic acid, glyceric acid, creatine, L-threonine, and citrulline.
