# Supplementary material for: A Serum Metabolite Classifier for the Early Detection of Type 2 Diabetes Mellitus-Positive Hepatocellular Cancer
Source: Metabolites. 2022 Jul 1;12(7):610. doi: 10.3390/metabo12070610 (PMC9315765; doi:10.3390/metabo12070610)
Supplement: Supplementary file 1 [file metabolites-12-00610-s001.zip › Table S1.pdf]

Table S1. MRM settings for benzoic acid, glyceric acid, creatine, L-threonine and citrulline

Q1 Mass (Da)	Q3 Mass (Da)	Dwell Time (msec)	ID	DP (volts)	EP (volts)	CE (volts)	CXP (volts)
120.9	76.9	25	Benzoic acid-1	-40	-10	-18	-15
120.9	93.0	25	Benzoic acid-2	-40	-10	-20	-15
104.9	75.0	25	Glyceric acid-1	-40	-10	-16	-15
104.9	57.0	25	Glyceric acid-2	-40	-10	-20	-15
176.0	159.2	25	Citrulline-1	52	10	15	15
176.0	113.3	25	Citrulline-2	52	10	23	15
132.0	90.2	25	Creatine-1	60	10	18	6
132.0	87.0	25	Creatine-2	60	10	24	6
120.1	73.9	25	L-Threonine-1	50	10	18	6
120.1	56.1	25	L-Threonine-2	50	10	24	6
120.1	84.0	25	L-Threonine-3	50	10	24	6
153.2	87.9	25	L-Glutamic acid -2,3,3,4,4-D5-1	40	10	20	8
153.2	135.1	25	L-Glutamic acid -2,3,3,4,4-D5-2	40	10	14	8
