# Supplementary material for: A Serum Metabolite Classifier for the Early Detection of Type 2 Diabetes Mellitus-Positive Hepatocellular Cancer
Source: Metabolites. 2022 Jul 1;12(7):610. doi: 10.3390/metabo12070610 (PMC9315765; doi:10.3390/metabo12070610)
Supplement: Supplementary file 1 [file metabolites-12-00610-s001.zip › Table S2.pdf]

Table S2. List of differential metabolites identified in the metabolomic analyses

Metabolite	MS2 score	SuperClass	VIP	P Value	FC	Log_FC
Homoarecoline	0.9836	Alkaloids and derivatives	1.6741	0.0001	0.7683	-0.3802
1,2-Dihydro-1,1,6-trimethylnaphthalene	0.8928	Benzenoids	2.7552	0.0000	0.5525	-0.8561
Benzaldehyde	0.9201	Benzenoids	1.6613	0.0076	1.5934	0.6721
2-Phenylethanol	0.9373	Benzenoids	2.1139	0.0001	1.6448	0.7179
Benzoic acid	0.9950	Benzenoids	2.6529	0.0001	2.0267	1.0192
2-Methylhippuric acid	0.6916	Benzenoids	1.7451	0.0043	2.1985	1.1365
Butylparaben	0.6733	Benzenoids	2.9261	0.0000	4.6136	2.2059
1-Methyl-1,3-cyclohexadiene	0.8244	Hydrocarbons	1.4331	0.0053	0.8999	-0.1522
Pregnanetriol	0.7622	Lipids and lipid-like molecules	2.0363	0.0000	0.4133	-1.2746
PC(18:4(6Z,9Z,12Z,15Z)/18:1(11Z))	0.7082	Lipids and lipid-like molecules	1.4334	0.0136	0.6198	-0.6901
N-Cyclopropyl-trans-2-cis-6-nonadienamide	0.9400	Lipids and lipid-like molecules	2.7859	0.0000	0.6872	-0.5411
6beta-Hydroxytestosterone	0.8066	Lipids and lipid-like molecules	1.2485	0.0389	0.7675	-0.3817
PC(22:5(7Z,10Z,13Z,16Z,19Z)/18:2(9Z,12Z))	0.7928	Lipids and lipid-like molecules	1.2568	0.0256	0.7891	-0.3417
PC(22:5(7Z,10Z,13Z,16Z,19Z)/16:0)	0.6836	Lipids and lipid-like molecules	1.5313	0.0114	0.8529	-0.2296
Pelargonic acid	0.9999	Lipids and lipid-like molecules	1.2747	0.0178	1.1232	0.1676
PC(22:5(7Z,10Z,13Z,16Z,19Z)/P-16:0)	0.8313	Lipids and lipid-like molecules	1.0545	0.0290	1.3449	0.4275
(E)-2,6-Dimethyl-2,5-heptadienoic acid	0.6134	Lipids and lipid-like molecules	1.5895	0.0005	1.3755	0.4600
PC(20:3(8Z,11Z,14Z)/20:1(11Z))	0.8495	Lipids and lipid-like molecules	1.0410	0.0146	1.3959	0.4812
PC(P-18:1(11Z)/22:5(4Z,7Z,10Z,13Z,16Z))	0.8300	Lipids and lipid-like molecules	1.2593	0.0026	1.3976	0.4829
PC(P-16:0/16:0)	0.6759	Lipids and lipid-like molecules	1.6758	0.0073	1.4797	0.5653
Azelaic acid	0.6424	Lipids and lipid-like molecules	1.3805	0.0056	1.4960	0.5812
PC(P-18:0/22:4(7Z,10Z,13Z,16Z))	0.8381	Lipids and lipid-like molecules	1.7603	0.0004	1.5029	0.5878
1-O-Hexadecyl-2-O-dihomogammalinolenoylglycero-3-phosphocholine	0.8612	Lipids and lipid-like molecules	1.9964	0.0010	1.6454	0.7184
PC(18:2(9Z,12Z)/P-18:1(11Z))	0.7329	Lipids and lipid-like molecules	1.7922	0.0003	1.6477	0.7204
3-Hydroxyisovaleric acid	0.9370	Lipids and lipid-like molecules	1.7349	0.0001	1.6689	0.7389
Ethyl oleate	0.7968	Lipids and lipid-like molecules	1.1517	0.0072	1.6872	0.7546

PC(18:0/P-16:0)	0.6660	Lipids and lipid-like molecules	2.3506	0.0003	1.7399	0.7990
16-Hydroxy hexadecanoic acid	0.9732	Lipids and lipid-like molecules	1.8678	0.0020	2.0424	1.0302
Octadecanedioic acid	0.6481	Lipids and lipid-like molecules	1.0454	0.0340	2.1652	1.1145
Ricinoleic acid	0.9862	Lipids and lipid-like molecules	1.4726	0.0454	2.6921	1.4288
15-Keto-13,14-dihydroprostaglandin A2	0.8173	Lipids and lipid-like molecules	1.6023	0.0457	2.7710	1.4704
Hexadecanedioic acid	0.9007	Lipids and lipid-like molecules	2.0187	0.0158	5.2489	2.3920
8,15-DiHETE	0.9765	Lipids and lipid-like molecules	1.5252	0.0114	7.9630	2.9933
N2,N2-Dimethylguanosine	0.9997	Nucleosides, nucleotides, and analogues	1.5517	0.0107	1.6611	0.7322
Creatine	0.9999	Organic acids and derivatives	1.6842	0.0000	0.6108	-0.7112
2-Ketobutyric acid	0.9970	Organic acids and derivatives	1.6742	0.0072	0.6134	-0.7052
Dimethylmalonic acid	0.7707	Organic acids and derivatives	1.3103	0.0300	1.1733	0.2305
L-alpha-Amino-1H-pyrrole-1-hexanoic acid	0.7357	Organic acids and derivatives	1.9796	0.0000	1.2544	0.3270
Betaine	0.9994	Organic acids and derivatives	1.7816	0.0027	1.2823	0.3588
Coutaric acid	0.7601	Organic acids and derivatives	1.4345	0.0024	1.3018	0.3805
L-Allothreonine	0.8758	Organic acids and derivatives	1.4867	0.0083	1.3063	0.3855
4-Guanidinobutanoic acid	0.9866	Organic acids and derivatives	1.8229	0.0001	1.3149	0.3950
L-Glutamine	0.6030	Organic acids and derivatives	1.1105	0.0370	1.3353	0.4172
L-Phenylalanine	0.9937	Organic acids and derivatives	2.2553	0.0001	1.3532	0.4363
Creatinine	0.9998	Organic acids and derivatives	1.7932	0.0084	1.3825	0.4672
Racemethionine	0.9576	Organic acids and derivatives	1.7791	0.0196	1.3906	0.4757
2-Hydroxyethanesulfonate	0.9459	Organic acids and derivatives	1.5437	0.0267	1.4502	0.5363
Pyroglutamic acid	0.9993	Organic acids and derivatives	2.3833	0.0002	1.4882	0.5736
4-Hydroxyproline	0.9951	Organic acids and derivatives	1.1415	0.0350	1.5815	0.6613
N-Acetylornithine	0.7652	Organic acids and derivatives	1.1535	0.0307	1.5949	0.6735
L-Threonine	0.8118	Organic acids and derivatives	1.2569	0.0051	1.8870	0.9161
N-Ethylglycine	0.9845	Organic acids and derivatives	2.1316	0.0006	1.9038	0.9289
Prolylhydroxyproline	0.7719	Organic acids and derivatives	1.6002	0.0060	1.9602	0.9710
L-cis-3-Amino-2-pyrrolidinecarboxylic acid	0.7412	Organic acids and derivatives	1.9618	0.0054	1.9989	0.9992
N-Acetylserine	0.8068	Organic acids and derivatives	2.0950	0.0024	2.0686	1.0486
Asymmetric dimethylarginine	0.8669	Organic acids and derivatives	2.2147	0.0006	2.1985	1.1365
Citrulline	0.9887	Organic acids and derivatives	2.3293	0.0003	2.4283	1.2800
3-Methoxy-4-hydroxyphenylethyleneglycol sulfate	0.6931	Organic acids and derivatives	2.1395	0.0076	3.6205	1.8562
Formiminoglutamic acid	0.8541	Organic acids and derivatives	2.2984	0.0038	4.0429	2.0154
Beta-Guanidinopropionic acid	0.9840	Organic nitrogen compounds	2.1540	0.0000	0.4479	-1.1587
5-Imino-2-methyl-1-cyclopenten-1-ol	0.9910	Organic nitrogen compounds	1.7411	0.0005	0.8679	-0.2044
L-Carnitine	0.9963	Organic nitrogen compounds	1.1583	0.0410	1.0836	0.1158

Choline	0.9998	Organic nitrogen compounds	1.8147	0.0003	1.2297	0.2983
N,N-Dimethylaniline	0.9042	Organic nitrogen compounds	2.0079	0.0002	1.6792	0.7478
1-Cyano-2-hydroxy-3-butene	0.7761	Organic oxygen compounds	1.6141	0.0001	0.8908	-0.1668
D-Ribose	0.9498	Organic oxygen compounds	1.3119	0.0169	1.1822	0.2414
4-Hydroxybenzaldehyde	0.9994	Organic oxygen compounds	1.4817	0.0085	1.3345	0.4163
Acetone cyanohydrin	0.9854	Organic oxygen compounds	1.2066	0.0427	1.3560	0.4393
myo-Inositol	0.8230	Organic oxygen compounds	1.9332	0.0028	1.4557	0.5417
1-(beta-D-Ribofuranosyl)-1,4-dihydronicotinamide	0.8983	Organic oxygen compounds	1.8225	0.0064	1.5352	0.6185
Glyceric acid	0.9378	Organic oxygen compounds	2.4864	0.0002	2.5316	1.3401
Neotrehalose	0.7753	Organic oxygen compounds	1.6924	0.0270	2.5741	1.3641
(R)-N-Methylsalsolinol	0.7120	Organoheterocyclic compounds	2.6351	0.0000	0.5765	-0.7947
2-Pyrrolidinone	0.9999	Organoheterocyclic compounds	2.6477	0.0000	0.6811	-0.5541
3-(2-Furanylmethylene)pyrrolidine	0.9884	Organoheterocyclic compounds	1.4830	0.0004	0.8318	-0.2658
Imidazole-4-acetaldehyde	0.8289	Organoheterocyclic compounds	1.6566	0.0029	1.1756	0.2334
Fagomine	0.7012	Organoheterocyclic compounds	1.3151	0.0088	1.2524	0.3247
2-Methyl-6-(2-propenyl)pyrazine	0.9719	Organoheterocyclic compounds	1.0955	0.0246	1.2858	0.3627
Pyrimidine	0.9832	Organoheterocyclic compounds	1.4426	0.0035	1.3196	0.4001
5-Ethyl-2,4-dimethyloxazole	0.9664	Organoheterocyclic compounds	1.5598	0.0271	1.3390	0.4211
3-Acetyl-2,7-naphthyridine	0.8143	Organoheterocyclic compounds	1.5609	0.0047	1.3815	0.4663
1H-Indole-3-carboxaldehyde	1.0000	Organoheterocyclic compounds	1.1514	0.0363	1.4206	0.5065
N1-Methyl-2-pyridone-5-carboxamide	0.9102	Organoheterocyclic compounds	1.0841	0.0429	1.4684	0.5543
1H-Indole-2,3-dione	0.9294	Organoheterocyclic compounds	1.8991	0.0004	1.5186	0.6027
(Â±)-Tryptophan	0.9851	Organoheterocyclic compounds	1.6951	0.0040	1.5223	0.6063
6-Chloro-N-(1-methylethyl)-1,3,5-triazine-2,4-diamine	0.8459	Organoheterocyclic compounds	1.6959	0.0043	1.5230	0.6069
3-Methylcytosine	0.9933	Organoheterocyclic compounds	1.9862	0.0007	1.5245	0.6083
Indole	0.8307	Organoheterocyclic compounds	1.9963	0.0002	1.6339	0.7083
Kynurenic acid	0.9360	Organoheterocyclic compounds	1.2858	0.0481	1.8600	0.8953
N-Nitroso-pyrrolidine	0.9846	Organoheterocyclic compounds	2.1917	0.0033	1.9712	0.9791
7-Methylguanine	0.8188	Organoheterocyclic compounds	2.2912	0.0004	2.0083	1.0060
Caffeine	0.6022	Organoheterocyclic compounds	1.0568	0.0051	2.0505	1.0360
1-Methylhypoxanthine	0.9909	Organoheterocyclic compounds	1.7722	0.0146	2.0913	1.0644
3-Methylguanine	0.9758	Organoheterocyclic compounds	2.4064	0.0011	2.2206	1.1509
Tripropylamine	0.9724	Organonitrogen compounds	2.4715	0.0000	9.7596	3.2868
4-Acetyl-3-methylpyridine	0.7963	Organooxygen compounds	1.6660	0.0492	2.3556	1.2361
Isopentyl mercaptan	0.8542	Organosulfur compounds	2.1146	0.0005	2.7692	1.4694
Phenyllactic acid	0.9734	Phenylpropanoids and polyketides	1.6300	0.0068	2.6467	1.4042

PC(22:6(4Z,7Z,10Z,13Z,16Z,19Z)/16:0)	0.8826		1.7247	0.0035	0.7779	-0.3623
PC(P-18:1(9Z)/16:0)	0.8389		1.4243	0.0078	1.5347	0.6179
PC(P-18:1(9Z)/16:1(9Z))	0.8471		1.7770	0.0008	1.5982	0.6764
PC(o-18:1(9Z)/20:4(8Z,11Z,14Z,17Z))	0.8598		1.8609	0.0008	1.6008	0.6788

Abbreviations: VIP, variable importance in the projection; FC, fold change.
