# Supplementary material for: A Serum Metabolite Classifier for the Early Detection of Type 2 Diabetes Mellitus-Positive Hepatocellular Cancer
Source: Metabolites. 2022 Jul 1;12(7):610. doi: 10.3390/metabo12070610 (PMC9315765; doi:10.3390/metabo12070610)
Supplement: Supplementary file 1 [file metabolites-12-00610-s001.zip › Table S3.pdf]

Table S3. The results of methodology validation for the quantification of serum benzoic acid, glyceric acid, creatine, L-threonine and citrulline

	Precisions				Linear Range	Regression Coefficient (R^2)	Recoveries	
	intra-day		inter-day					
	Low	High	Low	High			Low	High
Benzoic acid	5.32%	3.24%	11.28%	8.85%	1.5625-100 μM	0.999	108.68%	95.52%
Glyceric acid	8.25%	5.12%	6.51%	6.37%	3.125-100 μM	0.99	107.79%	92.73%
Creatine	2.42%	4.26%	11.38%	8.96%	6.25-200 μM	0.99	106.84%	103.37%
L-Threonine	4.86%	1.57%	12.85%	6.98%	12.5-400 μM	0.999	104.68%	100.41%
Citrulline	7.96%	5.10%	5.49%	3.83%	0.625-20 μg/ml	0.99	103.53%	107.05%
