# Supplementary figures and images for: A Serum Metabolite Classifier for the Early Detection of Type 2 Diabetes Mellitus-Positive Hepatocellular Cancer
Source: Metabolites. 2022 Jul 1;12(7):610. doi: 10.3390/metabo12070610 (PMC9315765; doi:10.3390/metabo12070610)

A

RT: 0.00 - 12.02

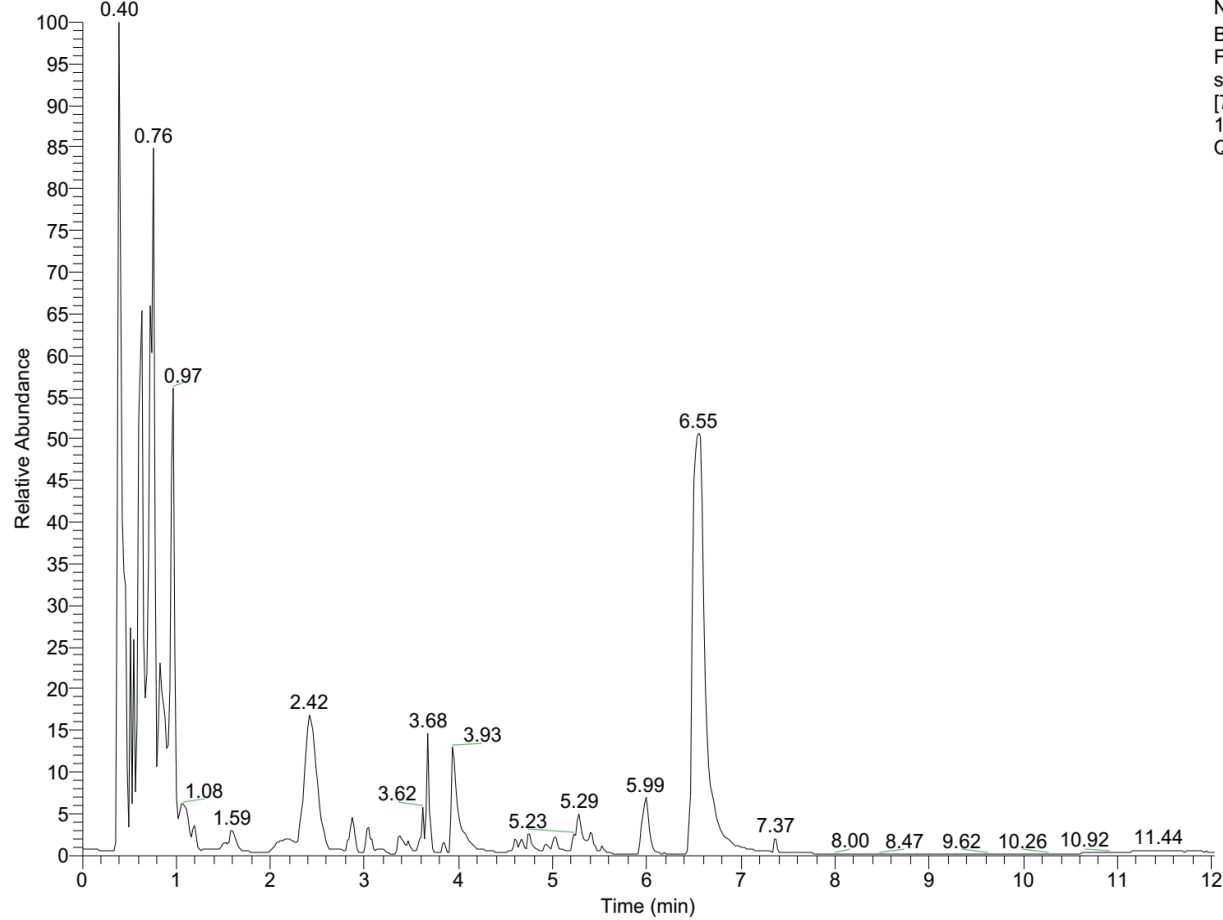

NL: 1.67E9  
Base Peak F:  
FTMS - p ESI  
sid=5.00 Full ms  
[70.0000-  
1050.0000] MS  
QC01

B

RT: 0.00 - 12.02

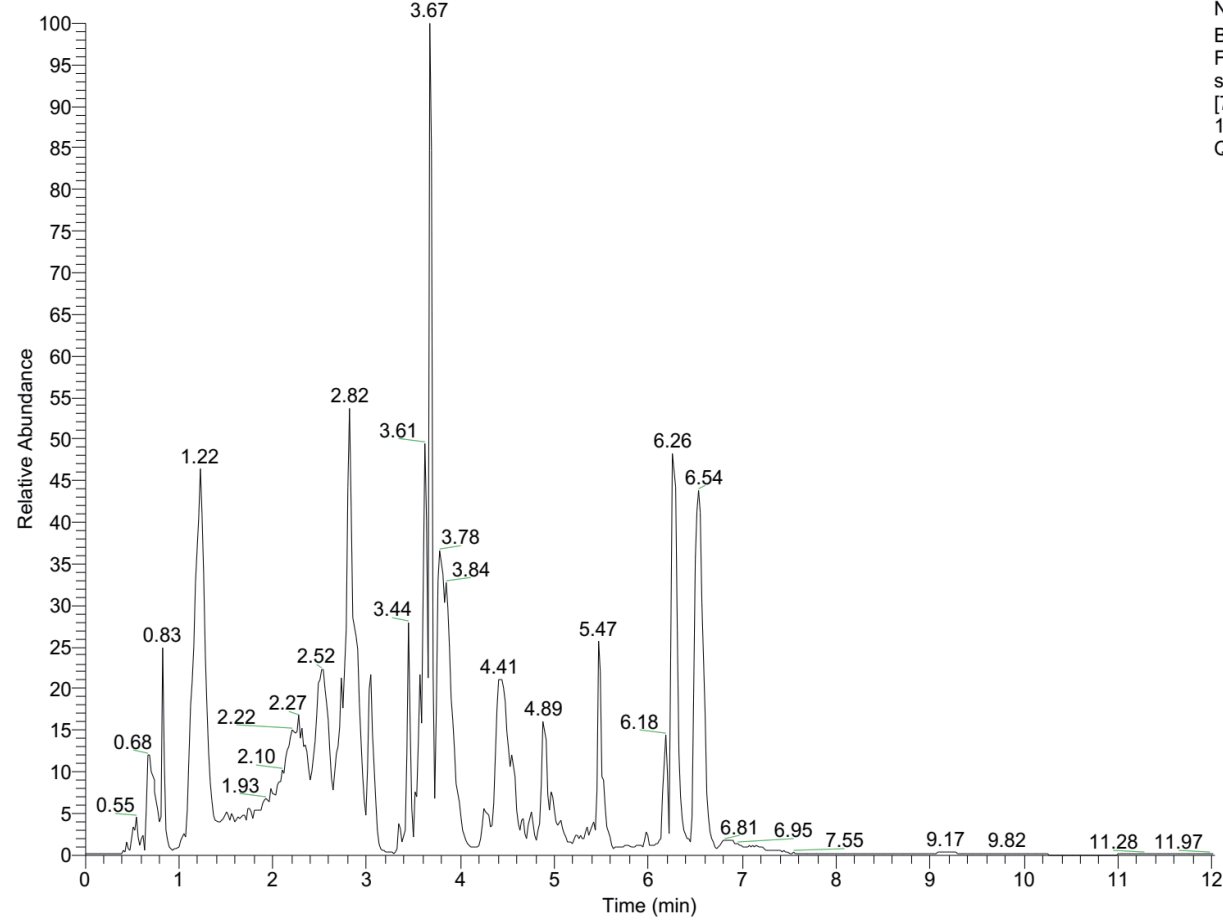

NL: 1.96E9  
Base Peak F:  
FTMS + p ESI  
sid=5.00 Full ms  
[70.0000-  
1050.0000] MS  
QC01

Supplement: Supplementary file 1 [file metabolites-12-00610-s001.zip › Figure S1.pdf]

A

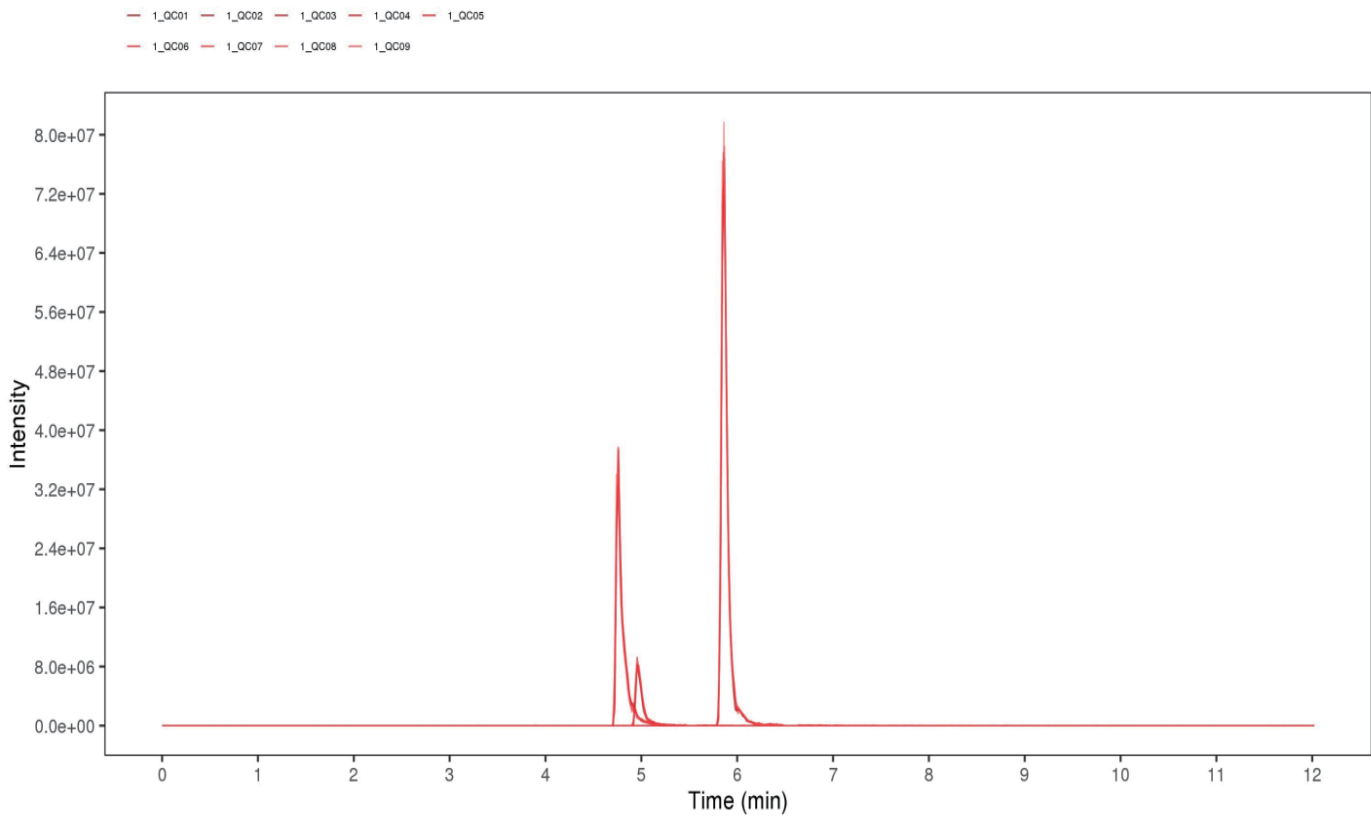

B

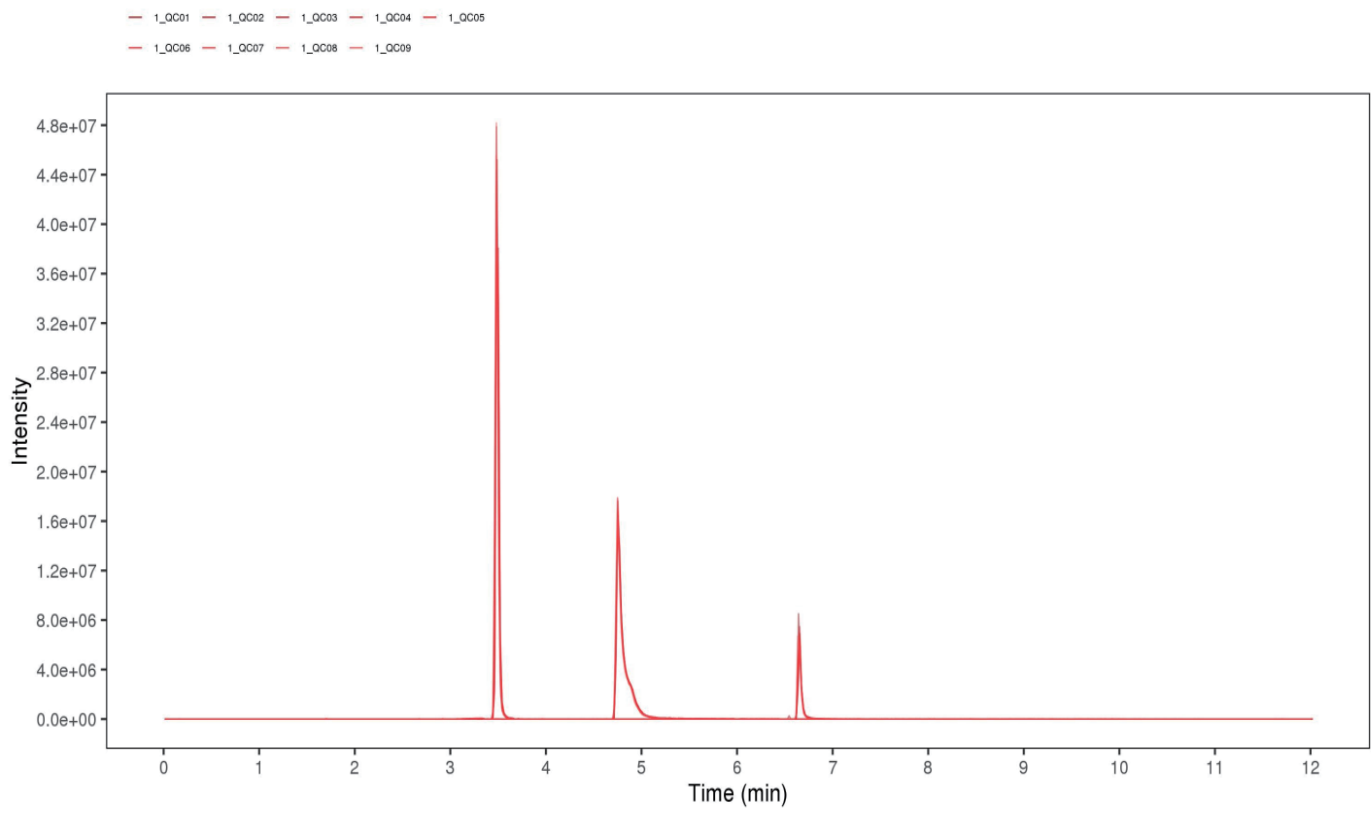

Supplement: Supplementary file 1 [file metabolites-12-00610-s001.zip › Figure S2.pdf]
